# Neoadjuvant Therapy (NAT) in Localized Pancreatic Cancer: Should We Do It and What Should We Do?

**DOI:** 10.33696/signaling.2.037

**Published:** 2021

**Authors:** Shreya Prasad Goyal, Morana Vojnic, Jung-in Yang, Jyothi Jose, Elliot Newman, M Wasif Saif

**Affiliations:** Northwell Health Cancer Institute, Lake Success, NY 11042, USA

**Keywords:** Chemotherapy, Neoadjuvant therapy, Pancreatic adenocarcinoma, Pancreatoduodenectomy, surgery, Gemcitabine, Oxaliplatin

In 2019, approximately 56,770 new cases of pancreatic cancer were diagnosed in the United States, resulting in an estimated 45,750 deaths. Pancreatic cancer is one of the leading causes of cancer-related death, with a five-year survival rate of 9% [1]. Based on the eighth edition of the American Joint Committee on Cancer (AJCC) staging system for pancreatic adenocarcinoma, multi-center analyses have validated that poorer prognosis is associated with node-positive disease (N1 and N2) [2,3]. Specifically, five-year survival rates were significantly lower with the increasing N stage: 35.6% in N0, 20.8% in N1, and 10.9% in N2, reflecting relatively better survival in organ confined pancreatic cancer, compared to node positive disease [3]. To date, the most effective treatment for pancreatic cancer is known to be surgical resection, partly due to the intrinsic resistance of pancreatic cancer cells to systemic therapy or radiotherapy. Unfortunately, only 15–20% of patients are candidates for surgical resection as most patients are diagnosed with locally advanced or metastatic disease, due to a lack of effective pancreatic cancer screening methods. However, the prognosis of pancreatic cancer is still grim even in those with resectable disease.

Complete resection of the neoplastic disease evident on imaging is far from curative treatment. Although patients with negative margins (R0) upon initial resection have significantly improved survival over those with positive margins (R1), re-resection of R1 pancreatic cancer to achieve final negative margin failed to improve survival, suggesting the mere technical aspects of clearing all local disease is not enough in this challenging disease. Moreover, approximately 72% of patients with R0 pancreatic cancer resections still had recurrence within two years. Notably, 77% of recurrence cases after complete resection presented with distant metastases [4, 5]. These facts emphasize that the problem in pancreatic cancer is not in what we *can* see on imaging, but in what we *cannot* see. Hence, the necessity of systemic therapy in patients with pancreatic cancer regardless of the disease stage. The proof of benefit for adjuvant therapy began with the CONKO-001 clinical trial, which revealed a significant benefit of adjuvant chemotherapy after R0 resection [6]. It is now clear that patients who receive adjuvant chemotherapy have a significantly decreased likelihood of recurrence when compared with patients who do not receive adjuvant therapy [7].

Accepting that pancreatic cancer is likely a systemic disease from the outset in the majority of patients, underscores the fact that systemic treatment may be the best first step. Patients tend to have a better functional status prior to invasive surgical procedures and better ability to tolerate treatments. Surgical complications or simply delayed recovery time after surgery can lead to as many as 25–45% of patients not receiving postoperative therapy [8,9]. This is especially important in the era of FOLFIRINOX therapy which is efficacious in the adjuvant setting, but more difficult to tolerate due to toxicity [10]. These factors have led to the use of neoadjuvant therapy (NAT) in patients with localized pancreatic cancer. In addition to tolerability, administering neoadjuvant chemotherapy allows an *in vivo* assessment of a tumor’s drug sensitivity, as well as potentially selecting patients who are most likely to benefit from surgery. Approximately 15% of patients can progress during NAT treatments [11,12]. One interpretation of this clinical finding is an aggressive disease biology which would not have been helped by immediate surgery. Alternatively, the question remains if a window of opportunity was lost in this group due to delay of surgical intervention with curative intent.

The benefits of multidisciplinary treatment in addition to surgery for pancreatic cancer have been demonstrated since the early 1980s, when the GITSG (Gastrointestinal Tumor Study Group) [13] concluded that the combined use of radiation therapy and fluorouracil as adjuvant therapy after curative resection is effective compared to no adjuvant therapy. Median survival in the control group was 10.9 months, compared with 21.0 months for those randomized to adjuvant treatment. Similarly, ESPAC-1 (European Study Group for Pancreatic Cancer 1 Trial) [14] randomized patients post-operatively to adjuvant fluorouracil-based chemoradiation alone, chemotherapy alone, chemoradiation and chemotherapy, or observation alone. This study, despite a complex study design, showed a survival benefit in the adjuvant chemotherapy arm (21% overall survival (OS) at 5 years, p=0.009), and no benefit to chemoradiation (10% OS at 5 years). Several other randomized trials have proven the benefit of adding systemic therapy to resected patients. The multinational CONKO-001 trial [6], conducted on patients with a complete resection who were randomly assigned to adjuvant gemcitabine or no treatment after surgery, showed a survival benefit from adjuvant gemcitabine. Patients randomized to adjuvant gemcitabine treatment had prolonged OS (5-year OS of 20.7%) compared with those randomized to observation alone (5-year OS 10.4%). While this study established single-agent gemcitabine as standard of care in the adjuvant treatment of pancreatic cancer, other subsequent studies proved combination treatments have even better efficacy. ESPAC- 4 [15] trial compared adjuvant gemcitabine in combination with capecitabine versus gemcitabine alone. The median OS for patients in the gemcitabine/capecitabine group was 28.0 months compared with 25.5 months in the gemcitabine group (p=0·032) showing the benefit of multi-agent therapy. The international APACT [16] trial showed the superiority of nab-Paclitaxel/gemcitabine combination versus gemcitabine alone for six cycles after a complete resection in patients with ECOG PS 0–1, showing a median OS 40.5 vs 36.2 months (p<0.05).

The other multidrug regimen that has been studied as an adjuvant to surgery is the FOLFIRINOX regimen. Recent data from the multicenter, open-label, phase 3 PRODIGE-24 trial [10] shows support of modified FOLFIRINOX (mFOLFIRINOX) compared to gemcitabine in adjuvant setting for resected pancreatic ductal adenocarcinoma. In this study, 493 patients who underwent complete (R0/R1) resections with a WHO performance status of 0–1 were randomly assigned to receive mFOLFIRINOX or gemcitabine. At a median follow up of 33.6 months, the median disease-free survival (DFS) was 21.6 months in the mFOLFIRINOX group compared to 12.8 months in the gemcitabine group. The secondary end point of the study was overall survival, with an OS of 54.4 months in the mFOLFIRINOX group and 35.0 months in the gemcitabine group.

With the clear benefit of additional therapy after surgery established, the question as raised above, of whether it is better to deliver these treatments before or after surgery was addressed in the PREOPANC trial [17]. This was a phase 3 clinical trial that randomly assigned 248 patients with potentially resectable or borderline resectable pancreatic cancer to upfront surgery followed by six months of adjuvant gemcitabine or to neoadjuvant gemcitabine-based chemoradiation followed by surgery and four months of adjuvant gemcitabine. The study population was about equally divided between resectable and borderline resectable patients. The R0 resection rate was 71% in patients who received preoperative chemoradiation and 40% in patients assigned to immediate surgery. However, the difference in median OS did not reach the level of statistical significance (16 vs 14.3 months). The Japanese Trial Prep-02/JASP-05 [18], randomized 362 patients with resectable only pancreas cancer to neoadjuvant gemcitabine plus S-1 followed by surgery versus upfront surgery. Both arms received six months adjuvant S-1 alone. The median OS in the neoadjuvant group was 36.7 months vs 26.6 months in upfront surgery group. Although grade 3 and 4 adverse events were observed frequently (73%) during preoperative chemotherapy, no significant differences for both groups were observed for perioperative outcomes. Importantly, the neoadjuvant arm noted a significant decrease of pathological nodal metastases and hepatic recurrence after surgery (30% vs 47.5%). Other support for the neoadjuvant approach comes from a large meta-analysis published in 2018 which compared upfront surgery versus NAT therapy in resectable and borderline resectable pancreas cancer. This paper specifically only included studies that reported data on an intention-to-treat basis and compared 1746 patients who had upfront surgery to 1738 patients who had NAT. There was a modest benefit in weighted mOS to the NAT group (18.8 months) compared to upfront surgery group (14.8 months) [19].

A shift towards neoadjuvant chemotherapy for resectable pancreatic cancer seems logical given the disease’s aggressive biology, the recurrence patterns as outlined earlier and some of the evidence cited above. These data suggest that pancreatic cancer should be considered a systemic disease, even when seemingly localized at diagnosis, and treated in such a manner. In addition, there is support for the potential benefits mentioned earlier. We know that in the PREOPANC trial almost 90% of patients completed preoperative therapy regimens, whereas only 62% of patients completed the adjuvant therapy in the preoperative arm and 53% completed adjuvant treatment in the immediate surgery arm, lending support to the notion that delivering the therapy preoperatively is more successful. Less than 3% of patients were unable to go to surgery because of treatment related toxicity or decline in performance status from preoperative therapy [17].

*Do we lose a window where surgical cure would have been possible*? This is not entirely clear, but the data from the studies cited above would seem to suggest that about 15% of patients did not come to surgery because of disease progression prior to surgery. In at least half of these cases the progression was metastatic in nature and the rest due to locally advanced disease [17].
*Is it possible with immediate surgery that this would not have occurred?* There is no way to answer this question definitively, but given the fact that half of the progression was metastatic in nature, it is likely these patients would have progressed soon after immediate surgery and that complex surgery would not have benefitted them. One can even make the argument that rapid local progression while receiving treatment indicates aggressive biology that would not have been amenable to curative surgery.
*Should we routinely use a NAT approach?* At this point in time, the answer is still unclear and not definitive. A lot of the data cited above includes both resectable and borderline resectable pancreas cancer. Should they be treated in a similar manner? Which definition of resectable and borderline disease should be used? There are a number of definitions that have been put forth to define resectable and borderline resectable. MD Anderson Cancer Center (MDACC), National Comprehensive Cancer Network (NCCN), International Study Group for Pancreatic Surgery (ISGPS), and Americas Hepatopancreaticobiliary Association/Society for Surgery of the Alimentary Tract/Society of Surgical Oncology (AHPBA/SSAT/SSO) all have published criteria with subtle differences related to terminology. However, the essence of what is being defined is the same. Resectable disease is a lesion confined to the pancreas where the likelihood of an R0 resection is very high given preserved planes around major vessels and the lack of vascular involvement. Conversely, borderline resectable defines a group of tumors where the likelihood of R0 resection is low given proximity to the major vasculature near the pancreas [20]. If we take a closer look at the PREOPANC data [17] specifically at the resectable versus borderline resectable patients, a subgroup analysis showed no difference in outcomes in the resectable group, but there was a benefit to receiving NAT in the borderline resectable group. This makes sense as achieving an R0 resection is harder in the borderline group given the definitions for these tumors outlined above. Given the fact that using neoadjuvant chemotherapy has the benefit of increasing the likelihood of an R0 resection [19] and its associated survival benefit [21], should only the borderline group receive this approach? Or as shown in the Prep02/JSAP-05 trial, should NAT be used in resectable disease as well [18]. Currently the NCCN guidelines for resectable disease have both immediate surgery and NAT as acceptable treatment options while recommending NAT for borderline resectable disease [22].
There are a number of ongoing clinical trials that are addressing this question and we can expect that when reported they will shed more light onto this clinical dilemma [23–26].
*What should we give?* This too remains unanswered. The SWOG S1505 phase II study looked at perioperative chemotherapy with either FOLFIRINOX or gemcitabine/nab-Paclitaxel for strictly resectable pancreatic adenocarcinoma and showed that patients can tolerate systemic therapy and undergo successful surgical resection. Of 103 eligible patients, 77 (75%) completed preoperative therapy and underwent surgery. Also, there was a 33% major pathologic response rate to preoperative therapy, either grade 0 tumor regression or grade 1 moderate response-minimal residual cancer [27]. The upcoming Alliance A02186 trial will examine a longer course of 8 cycles of neoadjuvant FOLFIRINOX in comparing perioperative to adjuvant FOLFIRINOX [28].
*What about radiation?* Its role in resectable or only borderline resectable, and type of fractionation, will also help determine the best course of treatment and how it should be administered.

As additional trials are conducted, it is important to address barriers to comparisons of outcomes in different approaches and with different regimens. One of the primary issues is the lack of uniform definitions for certain criteria. We already touched on the definitions or resectable and borderline resectable disease. Uniformity in definition is important in allowing for valid comparisons between studies. Second, there is not a consensus on the definition of an R0 resection. Third, pathologic examination protocols are not standardized [29, 30].

***In summary*, recent trends towards increased use of NAT in localized pancreas cancer is based on the bioloGy of the disease and the practical aspects of successfully deliverinG this therapy**. Emerging data suggests that NAT is associated with improved survival relative to upfront resection with respect to postoperative outcomes, oncologic outcomes, as well as overall survival in patients with localized pancreas cancer. However, definitive level one evidence for this paradigm shift is still lacking. In addition, whether this is applicable to both localized resectable disease and localized borderline resectable disease is also unclear. It is expected that the ongoing randomized clinical trials examining the use of neoadjuvant chemotherapy in these settings versus upfront surgery will further help overcome the selection bias in comparing outcomes between neoadjuvant and adjuvant trials and hopefully help us to develop the optimal treatment algorithm for to improve overall survival for one of the deadliest malignancies.
